# The chronic care model for type 2 diabetes: a systematic review

**DOI:** 10.1186/s13098-015-0119-z

**Published:** 2016-01-22

**Authors:** Deise Regina Baptista, Astrid Wiens, Roberto Pontarolo, Lara Regis, Walleri Christine Torelli Reis, Cassiano Januário Correr

**Affiliations:** Universidade Federal do Parana, Curitiba, Paraná Brazil

**Keywords:** Chronic care model, Chronic illness, Type 2 diabetes mellitus

## Abstract

The chronic care model (CCM) uses a systematic approach to restructure health care systems. The aim of this systematic review was to examine studies that evaluated different elements of the CCM in patients with type 2 diabetes mellitus (T2DM) and to assess the influence of the CCM on different clinical outcomes. There view was performed in the Medline and Cochrane Library electronic databases. The search was limited to randomized controlled trials conducted with T2DM patients. Studies were eligible for inclusion if they compared usual care with interventions that use done or more elements of the CCM and assessed the impact on clinical outcomes. After applying the eligibility criteria, 12 studies were included for data extraction. Of these, six showed evidence of effectiveness of the CCM for T2DM management in primary care as well as significant improvements in clinical outcomes. In the other six studies, no improvements regarding clinical outcomes were observed when comparing the intervention and control groups. Some limitations, such as a short follow-up period and a low number of patients, were observed. Some studies showed that the reorganization of health systems can improveT2DM care. However, it is possible that greater benefits could be obtained through combing all 6 elements of CCM.

## Background

Diabetes mellitus is currently a major chronic disease that affects individuals from countries at all stages of economic and social development. Even people in developed countries, despite scientific advances and easy access to health care systems, are affected by the increasing prevalence of diabetes [1–4].

The chronic care model (CCM) was developed to provide chronic disease patients, including those with type 2 diabetes mellitus (T2DM), with forms of self-care and tracking systems. The model represents a method for restructuring health care through interactions between health systems and communities [5]. In addition, the model collects basic data that can be used for improving care in health systems at the community, clinical practice, and patient levels [6–8].

The CCM, which was developed in the United States (USA) in 1990, synthesizes various components of disease management programs [9–11]. The CCM aims to improve and optimize six key, interrelated elements of the health system: organization of health care, self-management support, decision support, delivery system design, clinical information systems, and community resources and policies [8]. The essential focus of the model is to improve the use of existing resources, create new resources, and promote a new policy of interaction between more enlightened and empowered patients and better prepared and proactive health teams [6, 12].

Health services that are organized in a network and structured according to the CCM achieve better results in terms of completeness and resolution. Thus, incorporating the CCM in all levels of health care should be validated for feasibility in health systems in different countries [6, 7].

The aim of this study was to conduct a systematic review of randomized controlled trials (RCTs) evaluating different elements of the CCM and to assess their influence on clinical outcomes for patients with T2DM.

## Methods

A systematic review was performed searching the Medline and Cochrane Library databases, were used the describers: chronic care model, diabetes, chronic disease management, chronic illness model, chronic illness care, chronic illness management, chronic disease, chronic disease care and healthcare. The search was limited to RCTs that compared two groups of patients with T2DM: those in an intervention group, consisting of one or more elements of the CCM, and those in a control group, consisting of usual care for the pathology. Additional search was conducted by manual search and gray literature. Two reviewers conducted independent searches until May 2014 and included all articles published in English, Spanish, or Portuguese without restriction on publication date. If a lack of consensus between the two reviewers occurred, regardless of the stage of the study, a third reviewer was consulted.

RCTs were included if they were conducted over a 3 month period and evaluated the effects of the CCM on primary clinical outcomes (mortality) or intermediate clinical outcomes (HbA1c). RCTs were excluded if the articles that were not available in the full version (i.e., the abstract only), were clinical trials with patients with type 1 diabetes mellitus (T1DM), or evaluated patients less than 18 years old or patients with other chronic diseases. Articles describing the study protocol without presenting results and studies without clinical outcomes were also excluded.

Data related to the study duration, number of patients, study location, patient demographic characteristics, type of intervention conducted, and the CCM elements used were collected from the included studies. Data regarding how the implementation of the CCM affected the primary and intermediate clinical outcomes, beyond the conclusion of each study, were also extracted. Data were collected for further discussion.

## Review

**Fig. 1:**
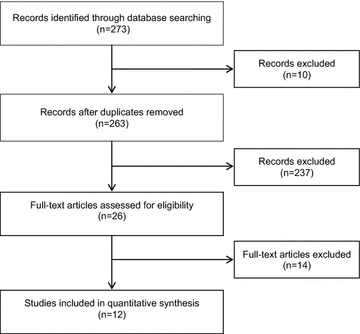
Systematic review flowchart for the chronic care model used in type 2 diabetes mellitus management

Based on the titles and abstracts, 273 studies were found and included for the first screening. Of these, 10 were duplicates and 237 articles were excluded based on the afore mentioned criteria (13 studies were not randomized, 160 evaluated other pathologies, 44 described only the protocol, one did not include clinical outcomes, three lasted less than 3 months, six were not available as full text (even after contact with the author), three evaluated T1DM, six did not evaluate the CCM, and one study was not completed). Therefore, 26 articles were included for thorough evaluation and, of these, 12 were included for data extraction (Fig. 1).

Characteristic of the included studies are presented in Table 1. Only one study reported blinding of both patients and data collectors [13] and one other study [14] reported blinding of patients only.

**Table 1:** Characteristics of included randomized control trials

References	Number of participants	Demographic characteristics (age and gender)	Study duration	Primary outcomes	Secondary outcomes
Wagner et al. [7]	707	Mean age of intervention group: 61.2 years Males in intervention group: 56 %	24 months	3 scales of SF-36 (general health, physical function, and physical role function), the presence of bed disability, and restricted-activity days	CES-D, HbA1c, total cholesterol, number of visits and hospitalization
Glasgow et al. [21]	886	Mean age of intervention group: 62 years Males in intervention group: 47.7 %	6 months	Improving both laboratory assays and patient-centered aspects of care	Quality of life, biologic outcomes (lipids and HbA1c levels), and depressive symptoms
Piatt et al. [16]	119	Mean age of intervention group: 69 years Males in intervention group: 53.3 %	1 year	HbA1c, LDL-C, and BP	Quality of life and glycemic self-monitoring
Hiss et al. [18]	197	Mean age of intervention group: 55.7 years Males in intervention group: 32 %	6 months	HbA1C, BP, and cholesterol	NR
Smith et al. [19]	639	Mean age of intervention group: 62 years Males in intervention group: 45 %	21 months (range 3–36 months)	The process of diabetes care, metabolic and vascular risk factor control, and the cost of care	BP, HbA1c, LDL-C, creatinine, and microalbumin levels
Goderis et al. [14]	2475	Mean age of intervention group: 68 years Males in intervention group: 47 %	18 months	HbA1c, SBP, and LDL-C levels	HDL-C, total cholesterol, DPB, weight, smoking status, statin and antiplatelet therapy efficacy
Schillinger et al. [17]	339	Mean age of participants: 56.1 years Males in intervention group: 41 %	1 year	1-year change in self-management behavior	HbA1C, SBP, DBP, and BMI
Glasgow et al. [23]	463	Mean age of intervention group: 58.7 years Males in intervention group: 55.4 %	4 months		HbA1c, BMI, lipids, and BP
Carter et al. [22]	47	Mean age of intervention group: 52 years Males in intervention group: 30.7 %	9 months	HbA1c level of 7 % or less	BP less than 130/80 and achieving a BMI between 18.5 and 24.9
Foy et al. [20]	869	NR	5 years	Mean HbA1c, cholesterol, and BP levels, and numbers of patients with recorded foot inspections in the previous calendar month	The number of patients within target ranges for HbA1c, cholesterol, and BP; the number of HbA1c, cholesterol, and ACR tests requested; and the mean practice BP levels for patients with and without recorded microalbuminuria
Lee et al. [13]	157	Mean age not reported Males in intervention group: 39.1 %	28 weeks	HbA1c concentration, DM self-efficacy scale, dietary behaviors, BMI, and waist circumference	Lifestyle changes (e.g., eating habits)
Piatt [15]	119	Mean age of intervention group: 69 years Males in intervention group: 53.3 %	3 years	Sustained improvements in HbA1C, non-HDL-C, and BP levels at 3 year follow-up	Diabetes knowledge, empowerment, quality of life, and self-monitoring of glycemia

Adapted from Wagner et al. [7]

*SF* short form health survey; *CES-D* center for epidemiologic studies depression scale; *HbA1c* glycated hemoglobin; *LDL-C* low-density lipoprotein cholesterol; *BP* blood pressure; *NR* not reported; *SBP* systolic blood pressure; *HDL-C* high-density lipoprotein cholesterol; *DBP* diastolic blood pressure; *BMI* body mass index; *ACR* albumin/creatinine ratio; *DM* diabetes mellitus

Regarding the study setting, two studies were conducted at private clinics [15, 16] in Pennsylvania, USA. Two studies were in community health clinics, one in San Francisco, USA [17] and one in Michigan, USA [18]. One study was conducted in a health maintenance organization in the USA [7], another in general outpatient clinics in Hong Kong [13], and the remaining six were conducted in primary care clinics [14, 19–23].

The average age of participants in the intervention group ranged between 52 and 69 years, although age was not reported in two studies [13, 20], and 32–56 % of patients in the intervention groups were male [20]. The duration of the interventions ranged from 4 months to 5 years.

## CCM elements

### Organization of health care services

**Fig. 2:**
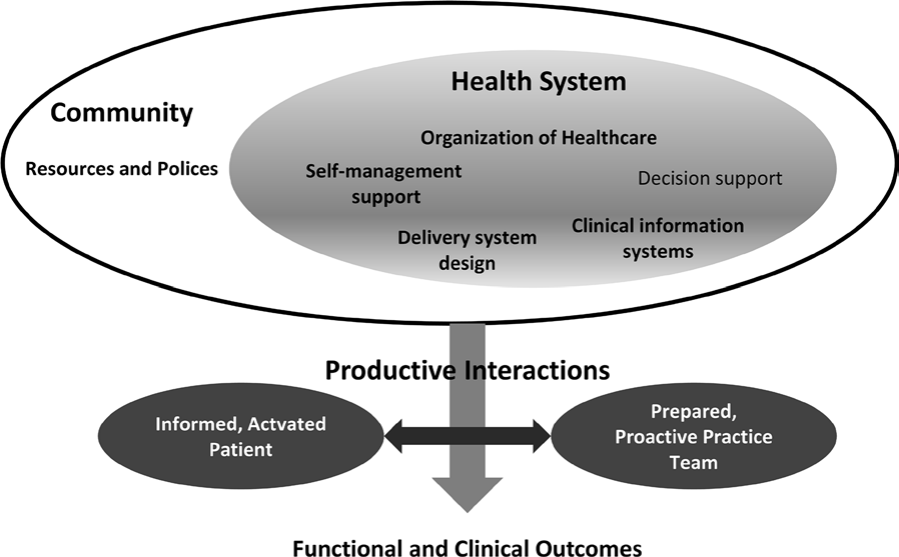
The chronic care model

The implementation of changes to the CCM (Fig. 2) by leaders in each health care organization was considered a health priority and a vital part of each organizations’ strategic plans in all studies [14–24]. The organization of health care services should focus on creating a culture and mechanisms that promote safe, high quality care. To enhance health care, improvements to service organization, introduction of strategies to facilitate changes, and management of errors and quality control problems are also necessary. Problems of miscommunication and coordination of health care must be prevented through agreements that facilitate communication and the flow of information between managers and service providers. Effective care for chronic conditions is virtually impossible without an information system to ensure ready access to key data from populations, subpopulations, and individuals [24–28].

### Self-care support

Ten studies addressed health service user empowerment for patients who self-manage their health care [7, 14–18, 21–23]. Interventions targeted user empowerment by emphasizing the role of users in managing their own health, the use of support strategies for self-care (including assessment of health status), goal setting, plan of care preparation and monitoring. The interventions were examined for recognition of the central role of users in their health care and development of a sense of self-responsibility related to health including regular use of evidence-based support programs that provided information, emotional support, and strategies for living with chronic conditions. Both the patient and provider should be included in defining problems, setting priorities, proposing goals, developing care plans, and monitoring results for self-care. Health professionals should prioritize collaborative care management so that prescribers become partners with health care system users [25, 27–31].

### Clinical decision support

Changes to clinical decision support promoted consistent attention in everyday practice of health care systems through the introduction of scientifically based clinical guidelines. In the 12 studies evaluated, changes in the behavior of health professionals were observed [13–24]. To increase user understanding, clinical decisions should be discussed and made together with the users. To change practices, clinical guidelines should include system alerts, reminders, and feedback [19, 25, 26, 28, 32–34].

### Clinical information systems

Changes to clinical information systems were observed in all of the studies analyzed. These changes aimed to organize user data to facilitate the efficiency and effectiveness of the health care system [7, 13–23, 35]. In these studies, user data was organized through an information system was used to facilitate attention to health, thus, making the information system more efficient and effective. Alerts, reminders, and timely feedback for health professionals as well as service users should be used when organizing user data. Organization of user data should also summarize clinical information to help identify risk groups that require different health care approaches and to allow for the monitoring of system performance and efforts made in order to provide better service quality [25, 27–31, 33, 34].

### Design of the service delivery system

Service provisions that would ensure attention to effective and efficient health care with transformation of the health system were observed in all of the 12 studies included in the systematic review [13–23, 35]. Improving the health of people with chronic conditions requires transforming a health care system that is essentially reactive, episodic, events focused, and responds to demands and acute conditions into a system that is proactive, integrative, continuous, and focuses on the person and family and is devoted to promoting and maintaining health. This requires that health care needs as well as roles and tasks be defined to ensure that users receive structured attention that is planned and provided by a multidisciplinary team. It means introducing new forms of care that go beyond face-to-face consultation (as a means of shared attention away from groups) to sustained attention, peer attention, and attention from a distance. The objective is to increase the amount of calls scheduled in advance to ensure that these calls are not made through spontaneous demand [24, 25, 27, 28, 31–34].

### Community resources

The community resources element of the CCM aims to mobilize resources to meet the needs of users through community programs and partnerships between health organizations and community organizations. The goal of this element is to develop programs that benefit users and improve health care policies [28]. However, this element was not found in any of the 12 studies included in the systematic review.

### Main clinical outcomes

In six studies, no improvements in clinical outcomes were found between the intervention group and the usual care group [7, 14, 17, 19, 21, 23]. Results of these studies are discussed in further detail below.

Wagner et al. compared a usual care program with standardized assessments, visits with the primary care physician, nurse, and clinical pharmacist, and a group education/peer support meeting. After 24 months of intervention, there was no significant difference in HbA1c and total cholesterol between the two groups.

Glasgow et al. [21] compared standard care with an interactive computer-based program. The first part of the program focused on the medical care participants were receiving for diabetes while the second part focused on development of a self-management action plan. Intervention patients answered questions regarding their dietary habits, physical activity, and smoking behaviors and then received feedback in each of these areas. Next, participants selected a behavior change goal in the area of smoking, diet, or exercise. After 6 months, both the control and intervention participants showed improved lipid and HbA1c levels, but there was no significant difference between the two groups.

In the 2008 study conducted by Smith et al. [19], those receiving a telemedicine intervention, which provided specialized advice and evidence-based messages regarding medication management for cardiovascular risk, were compared with those not receiving an intervention. After an average of 21 months (range 3–36 months), blood pressure (BP), HbA1c, low-density lipoprotein cholesterol, creatinine, and microalbumin levels were compared between the groups; however, the authors found that the intervention did not significantly enhance metabolic outcomes when compared with control.

Goderis et al. [14] assessed improvements in high-density lipoprotein cholesterol (HDL-C), total cholesterol, diastolic blood pressure (DBP), weight, and smoking status, as well as statin and antiplatelet therapy efficacy between a usual care and an intervention group. The 18-month intervention focused on an intensified follow-up, shared care, and patient behavioral changes. No significant additional improvements were found for the outcomes in the intervention group when compared with control group.

In the Schillinger et al. study in 2009 [17], patients were assigned to one of three groups: (1) standard care, (2) an interactive weekly automated telephone self-management support with nurse follow-up intervention, or (3) monthly group medical visits from a physician with health educator facilitation. Clinical outcomes, such as glycemic control, HbA1c, systolic blood pressure (SBP), DBP, and body mass index (BMI), were assessed after 9 months. Glycemic control improved across all three arms, but there were no statistically significant differences in HbA1c, SBP, DBP or BMI change across the three groups.

In the Glasgow et al. study [23], one group received a self-administered, computer-assisted, self-management (CASM) program with personalized goals and action plans for medication taking, healthy eating, and BP while the other received the CASM program with social support (i.e., follow-up calls from intervention personnel) and was invited to attend a group session. Both groups were compared against the usual care group. No significant differences were found for the HbA1c, BMI, lipids, and BP outcomes between the groups at the 4-month follow-up.

In the remaining studies, improvements in at least one clinical outcome were reported in five papers, whereas in one study [16], the same patients were assessed again at the 3-year follow-up, but the data were reported elsewhere [15].

Piatt et al. [16], compared three groups: the first group received a CCM-based intervention that involved patient and provider education as well as other CCM elements in the community, the second group received only provider education in which patients attended one problem based learning session, and the third group received usual care. After 1 year, a decline in HbA1c and non-HDL-C levels was observed in the CCM-based intervention group but not in the other two groups. Improvements were also observed in the proportion of patients that self-monitored blood glucose and in HDL-C levels when compared with the other groups. No intervention effect was seen on BP levels. At the 3-year follow-up, improvements in glycemic and BP control as well as the proportion of participants who self-monitor their blood glucose that were found at the 12-month follow-up were sustained in the CCM group. At the 3-year follow-up, the CCM group also experienced greater improvements in A1C and non-HDL-C levels [15].

In the Hiss et al. study [18], the intervention group received individual counseling, problem identification, care planning, and management recommendations by a nurse care manager during 6 months. The intervention group was then compared with the group usual care. Significant improvements occurred in mean SBP and HbA1C levels for intervention group patients while there was a significant improvement in DBP only for patients in the clinical action-indicated group who had more than two contacts with the project nurse. No significant changes were found for cholesterol between groups.

In the Carter et al. study [22], usual care was compared with an intervention in which each participant was equipped with a laptop and peripherals that automatically transmitted patient data to the patient’s health record. Participants were required to use the peripherals to weigh themselves and check their BP weekly, and to monitor their blood glucose three times per day. Instructions were provided regarding how to access the portal and how to use the camera attached to the laptop for video conferencing with the project’s telehealth nurse. The analysis showed a significant association between participation in the intervention and achieving an HbA1c measure of 7 % or lower. A significant, positive relationship was also found between participation in the intervention and achieving a healthy BMI. However, no such association was found between being in the treatment group and maintaining BP at 130/80.

Foy et al. [20] tested an intervention in which healthcare professionals received brief educational messages added to both paper and electronic primary care practice laboratory test reports. Phase one messages, attached to HbA1c reports, targeted glycemic and cholesterol control. Phase two messages, attached to albumin/creatinine ratio reports, targeted BP control and foot inspection. Mean levels of HbA1c, cholesterol, and BP, and the number of patients with recorded foot inspections were assessed after 5 years. There was no intervention effect on HbA1c, good glycemic control, or mean cholesterol levels. Although there was no intervention effect on SBP, there was a mean annual reduction of 1.59 mmHg during the study period. However, there was a statistically significant mean annual reduction in DBP of 0.92 mmHg during the study period in the intervention group. There was also an increased likelihood of a recorded foot inspection in intervention participants.

In the study by Lee et al. [13], the experimental group underwent 6 weekly sessions of diabetes self-management with an emphasis on self-efficacy and a participatory approach. The experimental group was compared with the control group receiving usual care. In the experimental group, the proportion of subjects with normal HbA1c increased between the baseline survey and week 28 follow-up while no significant improvements were found in the control group at the 28-week follow-up. Significant differences were also found between the experimental and control groups regarding decreases in BMI.

### Limitations

The implementation of a CCM-based intervention, using any of the six elements, was expected to result in improved clinical outcomes for patients. However, improvements occurred in only six of 12 included studies, and several factors may have contributed to this. For example, given that most studies did not blind participants to their intervention status, patients may have had knowledge of their participation in a study. In addition, several studies reported trials that included follow-up periods that were too short [17, 19, 21, 23]. Other limitations described by the authors included self-report measures for behavior change [17, 23], small sample sizes [17, 21], inadequate training of study nurses [7], and the absence of a gold standard registry and electronic medical records data [21].

One limitation of this review is that only two databases were used for research However, this issue was mitigated since the included bases represent the largest and most important in health area.

## Conclusions

Prevention and early intervention associated with integrated management can be a multidimensional and systemic solution to the difficult and complex problem of how to provide care for chronic conditions, such as diabetes. Our review shows that the use of isolated components of CCM does not seem to be enough to improve clinical outcomes; however, it is possible that greater benefits could be obtained through interventions combining CCM’s six elements.
